# Conventional Cardiac Surgery in Donation After Circulatory Death Heart Transplantation: A United Network for Organ Sharing Registry Analysis

**DOI:** 10.1111/ctr.70569

**Published:** 2026-05-18

**Authors:** Samantha N. Machinski, Yeahwa Hong, Umar Nasim, Ander Dorken‐Gallastegi, Nidhi Iyanna, Brian E. Woolley, Gavin W. Hickey, Mary E. Keebler, Laura Seese, Edward T. Horn, David J. Kaczorowski

**Affiliations:** ^1^ Department of Cardiothoracic Surgery University of Pittsburgh Medical Center Pittsburgh Pennsylvania USA; ^2^ Department of Surgery University of Pittsburgh Medical Center Pittsburgh Pennsylvania USA; ^3^ Division of Cardiology University of Pittsburgh Medical Center Pittsburgh Pennsylvania USA; ^4^ Vascular Medicine Institute University of Pittsburgh Medical Center Pittsburgh Pennsylvania USA; ^5^ Children's Hospital of Pittsburgh University of Pittsburgh Medical Center Pittsburgh Pennsylvania USA; ^6^ Department of Pharmacy and Therapeutics University of Pittsburgh Medical Center Pittsburgh Pennsylvania USA

**Keywords:** conventional cardiac surgery, donation after circulatory death, heart transplant

## Abstract

**Objective:**

This study evaluates outcomes following donation after circulatory death (DCD) heart transplantation among recipients with prior conventional cardiac surgery.

**Methods:**

The UNOS registry was queried to analyze adult recipients who underwent DCD heart transplantation between 1/1/2019 and 3/31/2024. Patients with durable left ventricular assist devices or congenital heart surgery were excluded. Recipients were stratified by conventional cardiac surgical history prior to transplantation. The primary outcome was 1‐year posttransplant survival. Subgroup analyses investigated the impact of surgical subtype and donor type on 1‐year survival.

**Results:**

Among 785 DCD recipients included, 233 (29.7%) underwent prior conventional cardiac surgery. Conventional cardiac surgery was associated with reduced 1‐year survival (89.6% vs. 96.8%, *p* < 0.001). Inferior survival persisted in a propensity score‐matched comparison (89.0% vs. 95.3%, *p* = 0.02). Combined coronary revascularization and valvular interventions conferred the lowest 1‐year survival rates among surgical subtypes (77.9% vs. 96.8%, *p* < 0.001). Among adults with conventional cardiac surgery, DCD and DBD recipients demonstrated comparable 1‐year survival (aHR 0.897, 95% CI 0.57–1.40, *p* = 0.635).

**Conclusion:**

Conventional cardiac surgical history is associated with reduced early posttransplant survival. Within this subgroup, adjusted analyses demonstrated no observed difference in early outcomes between DCD and DBD donors. These findings suggest DCD heart transplantation may be a viable strategy for organ donor pool expansion in this high‐risk population.

**Practitioner Points:**

Growing transplant volume and waitlist demand underscore the importance of efficient, safe allograft allocation. Nearly one‐third of adults undergoing DCD heart transplant have undergone prior conventional cardiac surgery. Despite lower early survival among these recipients, outcomes are independent of donor type, suggesting that use of DCD donors in this population does not confer additional mortality risk and supporting the continued utilization of DCD donors in this high‐risk population.

**Abbreviations**
BTT: bridged to transplant
DBD: donation after brain death
CABG: coronary artery bypass grafting
DCD: donation after circulatory death
LVAD: left ventricular assist device
MCS: mechanical circulatory support
NRP: normothermic regional perfusion
OCS: organ care system
OHT: orthotopic heart transplantation
OPTN: organ procurement and transplantation network
aHR: adjusted hazard ratio
UNOS: united network for organ sharing.

## 1 Introduction

Orthotopic heart transplantation (OHT) is considered optimal therapy for select patients with end‐stage heart failure refractory to medical management. [1] Mismatch between organ supply and demand underscores the urgency to reduce waitlist mortality through efficient allocation and safe donor pool expansion. [2, 3] Technological advances in allograft procurement and preservation have facilitated increased utilization of hearts donated after circulatory death (DCD). [4, 5, 6] Current studies support the safety profile of DCD transplant, demonstrating posttransplant outcomes comparable to those of donation after brain death (DBD) recipients. [7, 8, 9, 10, 11] Evidence‐based recommendations for allograft allocation following brain death are strongly dependent on recipient characteristics. [12] Yet, available literature lacks clear criteria identifying key perioperative risk factors associated with adverse outcomes following DCD transplant.

Registry reports have demonstrated increasing numbers of recipients with conventional cardiac surgical history, a group consistently associated with substantially worse posttransplant outcomes. [13, 14, 15, 16, 17] Despite the rising acceptance of DCD transplantation and established risks associated with prior cardiac surgery, there is a paucity of data investigating how these two factors interact to influence posttransplant outcomes. To address this, our study aimed to (a) evaluate the impact of conventional cardiac surgery on posttransplant survival among DCD recipients, and (b) establish whether candidates with prior conventional cardiac surgery can safely undergo DCD heart transplantation with outcomes comparable to DBD.

## 2 Methods

### 2.1 Data Source

The United Network for Organ Sharing (UNOS) registry database was utilized for this study, containing prospectively collected data for all solid organ transplantation in the United States. Patient and medical center identifiers were excluded from analysis. This study was approved by the University of Pittsburgh Institutional Review Board (STUDY2025‐7‐178045, 7/17/2025). Informed consent was waived given the study's retrospective design.

### 2.2 Study Population

Adult recipients (age ≥ 18 years) who underwent isolated DCD heart transplantation from 1/1/2019 to 3/31/2024 were included. 1‐year follow‐up extended to 3/31/2025. Recipients supported with durable left ventricular assist devices (d‐LVADs) at listing were excluded to isolate the impact of prior cardiac surgery on outcomes. Recipients with congenital cardiac surgery were also excluded given their distinct operative course often involving multiple prior sternotomies and extensive vasculature reconstruction. Additionally, multi‐visceral and heterotopic heart transplant recipients, candidates who previously underwent heart transplantation. Recipients who could not be successfully linked to donor records were excluded from analysis. DBD recipients were excluded from the primary analysis but were included in subgroup analyses under the same inclusion and exclusion criteria. Recipients were stratified into two groups based on conventional cardiac surgical history as reported by UNOS.

### 2.3 Baseline Characteristics and Outcomes

Baseline demographic and outcome data were collected from UNOS registry database and compared between groups, including age, sex, race, body mass index, heart failure etiology, comorbidities, most recent pretransplant laboratory values, pretransplant intervention, donor‐related information, and transplant parameters. Baseline characteristic missingness was summarized in Table S1.

The primary outcome was 1‐year posttransplant survival. Secondary outcomes assessed hospital length of stay and rates of posttransplant renal failure requiring dialysis, stroke, permanent pacemaker insertion, and acute rejection requiring medical therapy.

Subgroup analysis assessed 1‐year posttransplant survival among cardiac surgical subtypes against no cardiac surgical history. This included comparisons between isolated coronary artery bypass grafting (CABG), isolated valvular replacement/repair, combined CABG and valvular replacement/repair, and “other” cardiac surgery as defined by UNOS. A separate sub‐analysis evaluated the impact of donor type on 1‐year survival of recipients with conventional cardiac surgery. This analysis included primary isolated DBD heart transplant recipients during the same study period and assessed all recipients with conventional cardiac surgical history who underwent DCD versus DBD transplant.

### 2.4 Statistical Analysis

Continuous variables were reported as mean ± standard deviation or median with interquartile range (IQR) and compared using Student's t‐test or Wilcoxon rank‐sum test, as appropriate. Categorical comparisons utilized Pearson's Chi‐square test. Normally distributed continuous variables and non‐normally distributed variables utilized Student's t‐test and Wilcoxon rank‐sum test, respectively. Unadjusted Kaplan‐Meier survival analyses with associated log‐rank testing evaluated the primary outcome. Cox proportional hazards regression assessed the impact of conventional cardiac surgery on posttransplant mortality after adjusting for established mortality risk factors. [18] Patients with missing data were excluded from multivariate modeling.

A propensity score‐matched analysis matched DCD recipients with and without conventional cardiac surgery to ensure similarities across baseline recipient, donor, and transplant characteristics. Matching was performed on a 1:1 basis utilizing nearest neighbor matching without replacement with caliper setting of 0.2 of the standard deviation of the logit propensity score. A standardized mean difference (SMD) was defined to be adequately matched or well‐matched at SMD <15% and <10%, respectively. Primary and secondary outcomes were analyzed in the propensity‐score‐matched cohort. All statistical analyses utilized Stata version 18.5 (StataCorp, College Station, TX).

## 3 Results

### 3.1 Baseline Recipient, Donor, and Transplant Characteristics

**FIGURE 1:**
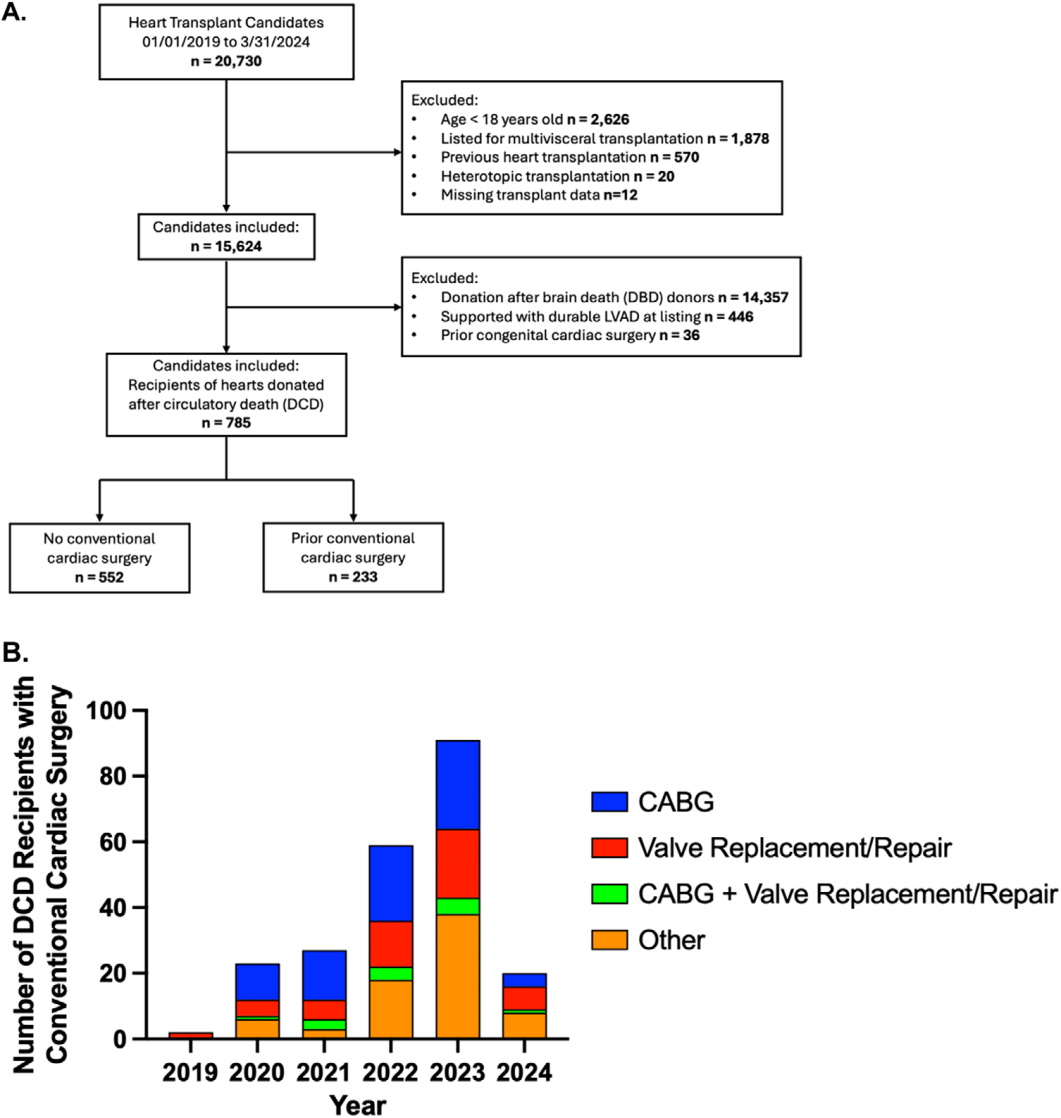
(A) Inclusion criteria. (B) Distribution of cardiac surgery subtypes in DCD heart transplant recipients with conventional cardiac surgical history.

Of 785 isolated DCD heart recipients included in this study, 233 (29.7%) underwent conventional cardiac surgery prior to transplantation (Figure 1A). On average, recipients with conventional cardiac surgery were older (61 vs. 57 years, *p* < 0.001), less often female (17.2% vs. 23.7%, *p* = 0.042), and more likely to have ischemic heart failure (43.3% vs. 22.6%, *p* < 0.001), diabetes (34.8% vs. 27.7%, *p* = 0.049), and require waitlist blood transfusion (24.5% vs. 8.3%, *p* < 0.001). The only observed difference in baseline donor characteristics was elevated total bilirubin (0.60 vs. 0.50 mg/dL, *p* = 0.046) for recipients with prior cardiac surgery. Recipients with conventional cardiac surgery were more likely to endure longer waitlist duration (34 vs. 23 days, *p* = 0.004) and graft preservation time (5.3 vs. 4.8 h, *p* = 0.02) (all Table 1).

**TABLE 1:** Baseline recipient, donor, and transplant characteristics with posttransplant outcomes and cause of death stratified by prior cardiac surgery.

Parameter	No Prior Cardiac Surgery (*n* = 552)	Prior Cardiac Surgery (*n* = 233)	*p*‐value
*Recipient Characteristics*			
Recipient age (years)	57 (45–64)	61 (52–66)	<0.001
Female gender	131 (23.7%)	40 (17.2%)	0.042
Body mass index (kg/m^2^)	27.68 (4.84)	28.38 (4.82)	0.062
Race			0.14
White	363 (65.9%)	174 (74.7%)	
Black	109 (19.8%)	31 (13.3%)	
Hispanic	61 (11.1%)	20 (8.6%)	
Asian	15 (2.7%)	6 (2.6%)	
Other	3 (0.5%)	2 (0.9%)	
Blood Type			0.69
A	204 (37.0%)	91 (39.1%)	
AB	20 (3.6%)	7 (3.0%)	
B	66 (12.0%)	33 (14.2%)	
O	262 (47.5%)	102 (43.8%)	
Education level			0.69
High school	206 (38.4%)	85 (37.0%)	
College	274 (51.0%)	115 (50.0%)	
Graduate degree	57 (10.6%)	30 (13.0%)	
Functional status			0.53
Independent	27 (5.2%)	16 (7.2%)	
Requires assistance	228 (43.6%)	97 (43.7%)	
Hospitalized	268 (51.2%)	109 (49.1%)	
Heart failure etiology			<0.001
Nonischemic	300 (54.3%)	77 (33.0%)	
Ischemic	125 (22.6%)	101 (43.3%)	
Congenital	5 (0.9%)	5 (2.1%)	
Restrictive	45 (8.2%)	7 (3.0%)	
Valvular	2 (0.4%)	20 (8.6%)	
Hypertrophic	35 (6.3%)	14 (6.0%)	
Other	0 (0.0%)	0 (0.0%)	
Intensive care unit state at time of transplantation	239 (43.3%)	82 (35.2%)	0.035
Pretransplant dialysis	6 (1.1%)	2 (0.9%)	0.77
Pretransplant mechanical ventilation	4 (0.7%)	3 (1.3%)	0.44
Pretransplant infection	31 (4.5%)	19 (6.6%)	0.19
Intravenous inotropes	232 (42.0%)	77 (33.0%)	0.019
Waitlist blood transfusion	46 (8.3%)	57 (24.5%)	<0.001
Cerebrovascular accident	38 (6.9%)	25 (10.7%)	0.070
Diabetes mellitus	153 (27.7%)	81 (34.8%)	0.049
Hypertension	130 (26.3%)	53 (25.7%)	0.88
Positive CMV serology	288 (52.2%)	116 (49.8%)	0.54
Total bilirubin (mg/dL)	0.70 (0.50–1.10)	0.60 (0.40–0.90)	0.007
Serum creatinine (mg/dL)	1.10 (0.9–1.36)	1.16 (0.95–1.40)	0.087
IABP	140 (25.4%)	39 (16.7%)	0.009
ECMO	14 (2.5%)	4 (1.7%)	0.48
Cardiac output (L/min)	4.30 (1.31)	4.29 (1.18)	0.96
Cardiac index (L/min/m^2^)	2.070147 (0.5962007)	2.030106 (0.4815583)	0.37
Mean pulmonary artery pressure (mmHg)	28.06 (10.18)	26.95 (10.84)	0.18
Mean pulmonary capillary wedge pressure (mmHg)	18.80 (8.70)	17.74 (8.31)	0.12
Mean pulmonary vascular resistance (Wood units)	2.48 (1.62)	2.33 (1.47)	0.24
Transpulmonary Gradient (mmHg)	9.66 (5.11)	9.25 (4.88)	0.33
*Donor Characteristics*			
Donor Age	31 (25–37)	31 (24–38)	0.57
Female gender	105 (19.0%)	36 (15.5%)	0.23
Body mass index	27.64 (6.50)	27.83 (6.32)	0.71
Donor race			0.93
White	409 (74.1%)	174 (74.7%)	
Black	52 (9.4%)	23 (9.9%)	
Hispanic	75 (13.6%)	28 (12.0%)	
Asian	6 (1.1%)	4 (1.7%)	
Other	10 (1.8%)	4 (1.7%)	
Donor blood type			0.83
A	230 (41.7%)	82 (35.2%)	
AB	2 (0.4%)	1 (0.4%)	
B	47 (8.5%)	22 (9.4%)	
O	323 (58.5%)	128 (54.9%)	
Donor mechanism of death			0.24
Trauma	276 (39.8%)	98 (42.1%)	
Cardiovascular	51 (9.2%)	21 (9.0%)	
Drug overdose	112 (20.3%)	60 (25.8%)	
Other	159 (28.8%)	54 (23.2%)	
Donor diabetes mellitus	12 (2.2%)	9 (3.9%)	0.18
Donor Hepatitis C	44 (8.0%)	21 (9.0%)	0.63
Positive CMV serology	311 (57.2%)	131 (56.2%)	0.81
Hypertension	65 (11.8%)	33 (14.3%)	0.33
Graft LVEF	63 (60–66.1)	64 (60–67)	0.39
Total bilirubin (mg/dL)	0.60 (0.40–1.00)	0.5 (0.40–0.90)	0.046
Serum creatinine (md/dL)	0.76 (0.60–1.04)	0.75 (0.60–1.00)	0.83
*Transplant Characteristics*			
Sex matched	452 (81.9%)	203 (87.1%)	0.071
Race matched	309 (56.0%)	142 (60.9%)	0.20
HLA matched	53 (9.6%)	22 (9.4%)	0.94
ABO matched	475 (86.1%)	202 (86.7%)	0.81
CMV status matched	300 (55.1%)	118 (50.6%)	0.25
Waitlist time (days)	23 (7–109.5)	34 (12–167)	0.004
Distance‐recipient distance (Nautical Miles)	307 (125.5–555)	342 (130–553)	0.82
Total graft ischemic time (hours)	4.8 (3.3–6.2)	5.3 (3.7–6.7)	0.02
Waitlist status at listing			0.61
1	9 (1.7%)	4 (1.8%)	
2	134 (25.3%)	44 (19.9%)	
3	40 (7.5%)	17 (7.7%)	
4	159 (30.0%)	64 (29.0%)	
5	2 (0.4%)	1 (0.5%)	
6	186 (35.1%)	91 (41.2%)	
Waitlist status at transplantation			0.44
1	17 (3.1%)	7 (3.0%)	
2	227 (41.1%)	88 (37.8%)	
3	59 (10.7%)	24 (10.3%)	
4	124 (22.5%)	69 (29.6%)	
5	2 (0.4%)	1 (0.4%)	
6	123 (22.3%)	44 (18.9%)	
*Posttransplant Complications*			
Dialysis	85 (15.4%)	49 (21.1%)	0.052
Stroke	14 (2.5%)	14 (6.0%)	0.017
Permanent pacemaker implantation	10 (1.8%)	2 (0.9%)	0.32
Length of stay (days)	15 (12–23)	16 (12–24)	0.44
Treated acute rejection	48 (8.7%)	15 (6.4%)	0.29
*Posttransplant Cause of Death*			
Graft Failure	7 (20%)	5 (14%)	0.44
Infection	7 (20%)	8 (22%)	
Cardiovascular	5 (14%)	3 (8%)	
Pulmonary	1 (3%)	1 (3%)	
Cerebrovascular	1 (3%)	6 (16%)	
Hemorrhage	0 (0%)	1 (3%)	
Malignancy	2 (6%)	5 (14%)	
Other, misc.	9 (26%)	7 (19%)	
Unknown/not reported	3 (9%)	1 (3%)	

**Abbreviations**: CMV, cytomegalovirus; ECMO, extracorporeal membrane oxygenation; HLA, human leukocyte antigen; IABP, intra‐aortic balloon pump; ICU, intensive care unit; LVEF, left ventricular ejection fraction.

### 3.2 Impact of Conventional Cardiac Surgery on Posttransplant Survival

**FIGURE 2:**
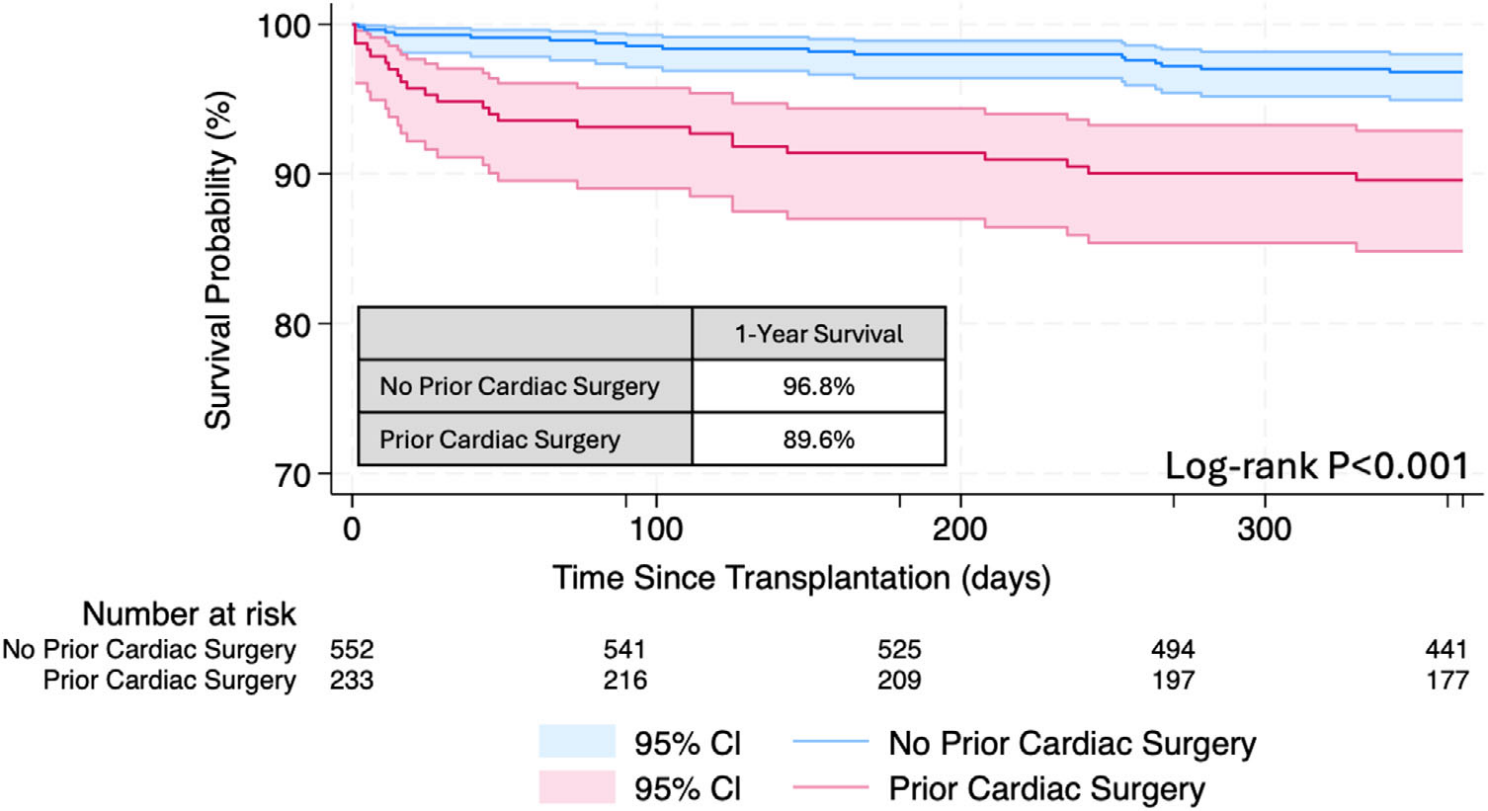
1‐year posttransplant survival following DCD heart transplantation stratified by conventional cardiac surgery.

Unadjusted Kaplan‐Meier analysis demonstrated lower 1‐year survival for DCD recipients with conventional cardiac surgical history compared to those without (89.6% vs. 96.8%, *p* < 0.001; Figure 2). DCD recipients with prior cardiac surgery had higher rates of posttransplant stroke (6.0% vs. 2.5%, *p* = 0.017) but comparable hospital length of stay and rates of posttransplant renal failure requiring dialysis, permanent pacemaker insertion, and acute rejection requiring medical therapy (Table 1). Reduced 1‐year survival with conventional cardiac surgery persisted following multivariable adjustment (aHR 3.26, 95% CI 1.71–6.22, *p* < 0.001) (Table S2). [18]

### 3.3 Propensity Score‐Matched Comparisons

**FIGURE 3:**
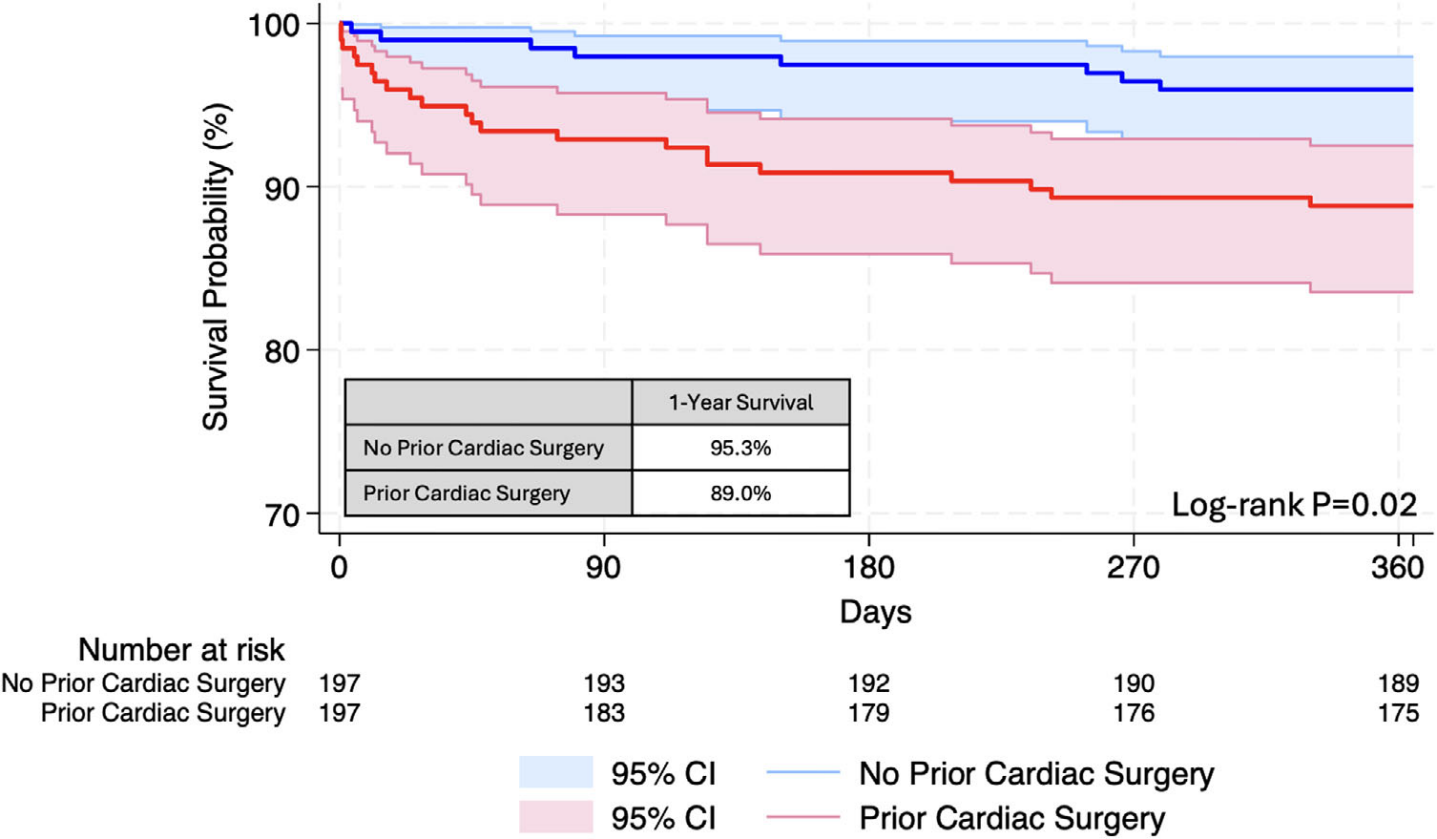
1‐year posttransplant survival following DCD heart transplantation stratified by conventional cardiac surgery in propensity score‐matched cohort.

Propensity score matching yielded 382 recipients of DCD hearts, including 191 with and without conventional cardiac surgery. Groups were adequately matched (Table S3A) except for differing heart failure etiology (*p* < 0.001) and longer waitlist time for recipients with prior cardiac surgery (34 vs. 21 days, *p* = 0.02). Conventional cardiac surgery was associated lower 1‐year posttransplant survival (89.0% vs. 95.3%, *p* = 0.02; Figure 3). Posttransplant outcomes were comparable between recipients with and without conventional cardiac surgical history in the matched cohort (Table S3B, all *p* > 0.05).

### 3.4 Cardiac Surgical Subtype and Posttransplant Survival

**FIGURE 4:**
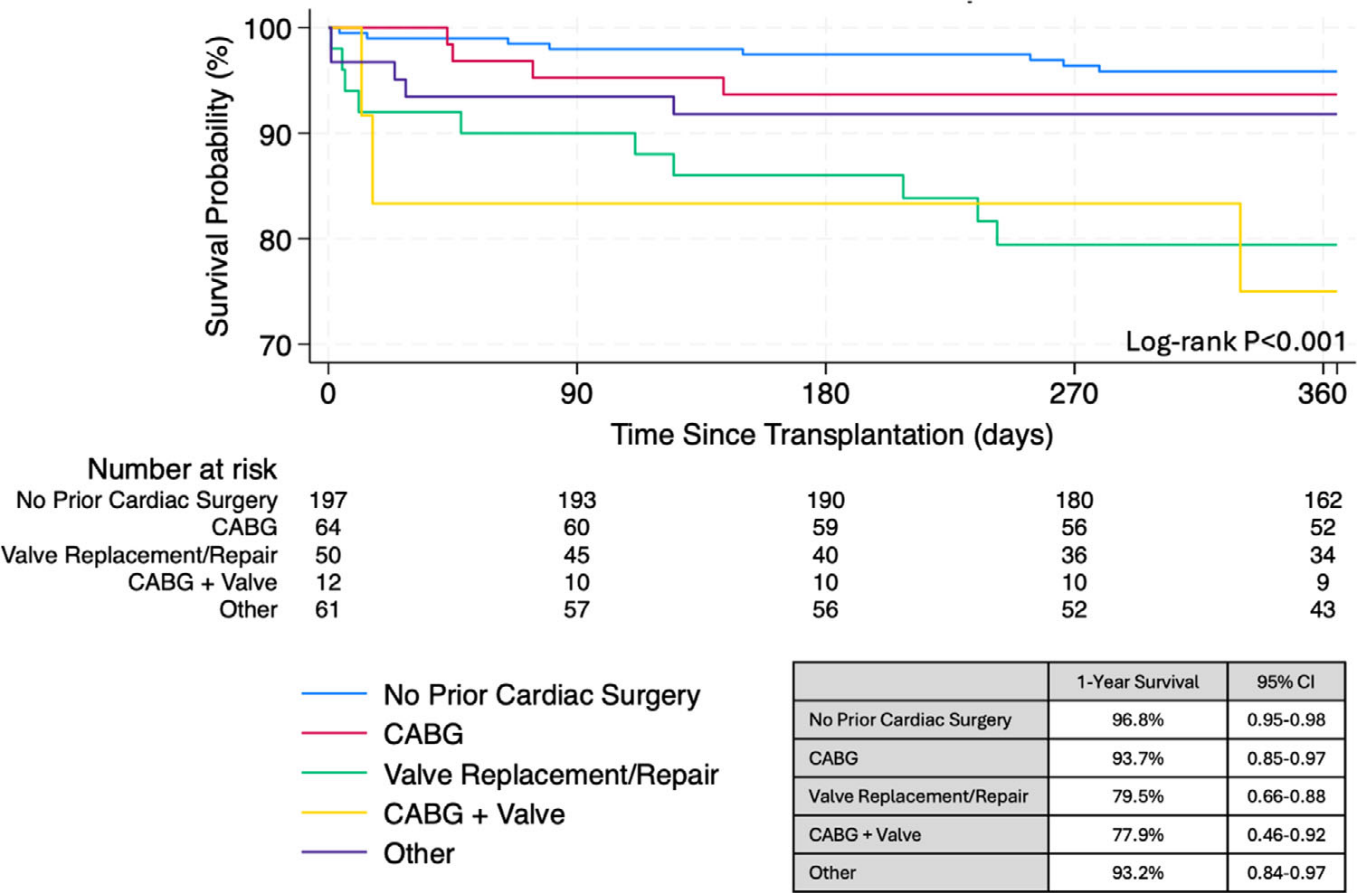
1‐year posttransplant survival following DCD heart transplantation stratified by conventional cardiac surgery subtype.

The incidence of DCD transplant recipients with conventional cardiac surgery has progressively increased since 2019 (Figure 1B). Among 233 total recipients with prior cardiac surgery, the most common procedures were isolated CABG (36.0%) and UNOS‐grouped “other” operations (32.9%). 1‐year posttransplant survival was significantly different across surgical subtypes (*p* < 0.001, Figure 4). Recipients with isolated valvular interventions (79.5%) or combined CABG and valvular interventions (77.9%) demonstrated the lowest 1‐year survival rates. Highest 1‐year survival was observed among recipients with isolated CABG (93.7%) compared to those without conventional cardiac surgery (96.8%).

### 3.5 DBD Versus DCD Transplantation in Recipients With Conventional Cardiac Surgery

**FIGURE 5:**
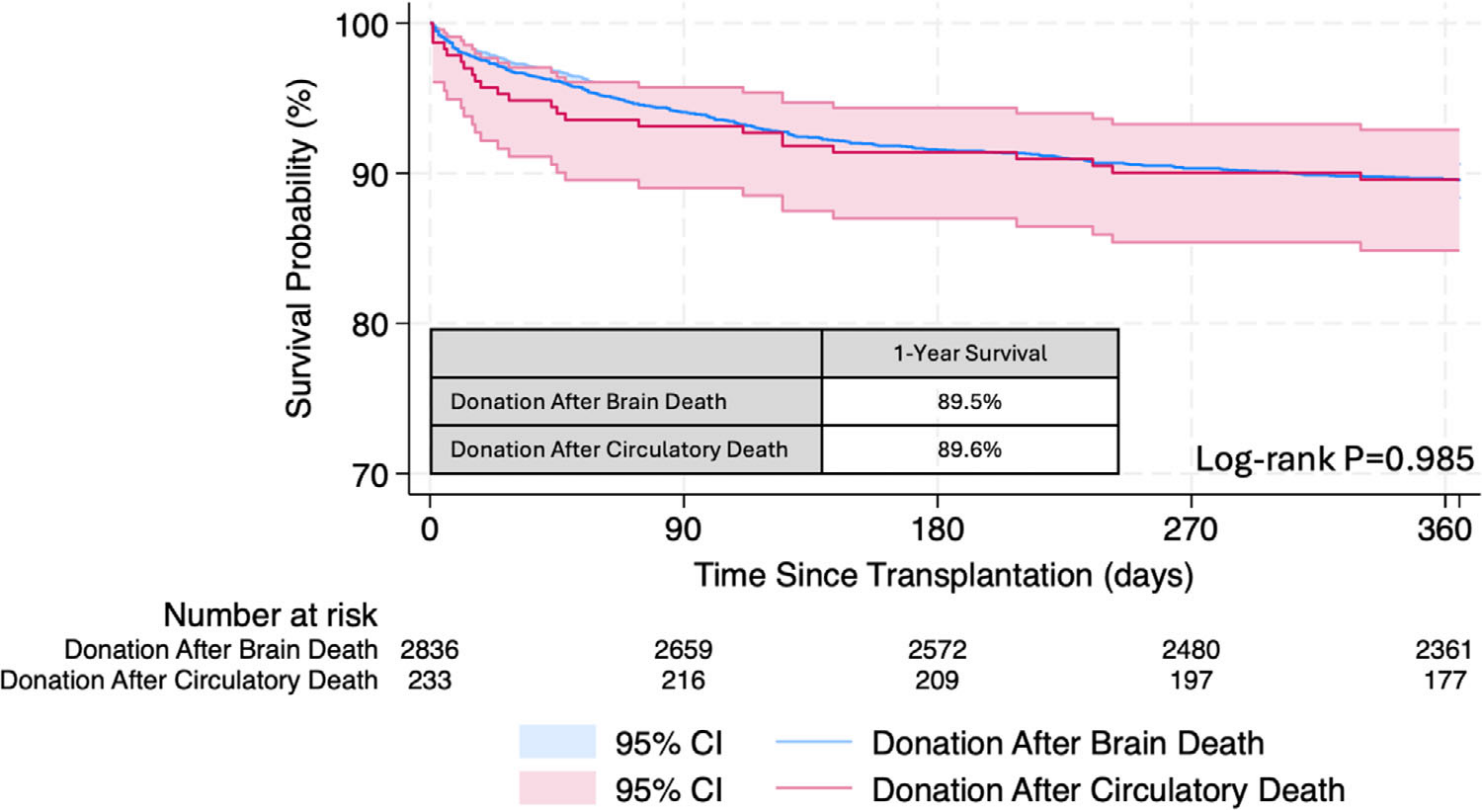
1‐year posttransplant survival of recipients with conventional cardiac surgery stratified by donor type (DCD vs. DBD).

Of 3069 total recipients with conventional cardiac surgical history, 2836 underwent DBD transplantation (92.4%) compared to 233 who underwent DCD transplantation (7.6%). Unadjusted Kaplan‐Meier survival demonstrated comparable1‐year survival among DCD and DBD recipients with conventional cardiac surgery (89.6% vs. 89.5%, *p* = 0.985; Figure 5). Comparable survival persisted after multivariable adjustment (aHR 0.897, 95% CI 0.57–1.40, *p* = 0.635) (Table S4). [18]

## 4 Discussion

This study utilized the UNOS registry to evaluate survival following DCD heart transplantation in recipients with prior conventional cardiac surgery, excluding those with d‐LVADs or congenital cardiac surgery. Notable findings from this analysis include (a) nearly one‐third of all isolated DCD recipients have undergone conventional cardiac surgery, (b) prior cardiac surgery is associated with substantially reduced 1‐year posttransplant survival, and (c) early posttransplant survival was comparable between DCD or DBD recipients with conventional cardiac surgery.

Evolving indications for heart transplant have enabled the inclusion of increasingly complex patients as viable candidates. Over the last three decades, the transplant population has observed a growing proportion of recipients with prior cardiac surgery (37.1% 1992–2000 vs. 43.6% 2001–2009 vs. 50.1% 2010–2018), [15] and nearly half (44.2%) of all heart transplant recipients between 1992 and 2024 have undergone cardiac surgery prior to transplant. [17] Our findings demonstrate similar prevalence, with 29.7% of DCD recipients having prior cardiac surgery. The slightly lower rate may reflect exclusion of recipients with congenital surgery or d‐LVADs. As this prevalence continues to rise, prior cardiac surgery will become an increasingly important factor in accurately defining recipient risk profiles.

Previous literature comparing transplant outcomes among recipients with and without conventional cardiac surgery have yielded inconsistent findings. Older reports utilizing smaller sample sizes indicate that prior cardiac operations do not compromise posttransplant survival. [19, 20, 21] However, these studies largely predate the widespread utilization of d‐LVADs and temporary MCS and therefore may not be generalizable to the older, more complex transplant population represented in contemporary clinical practice. A more recent, single‐center study found that conventional cardiac surgery was associated with inferior short‐ and long‐term survival and higher complication rates. [13] Additionally, non‐transplant cardiac surgery was associated with higher 1‐year posttransplant mortality risk by the ISHLT 2025 Annual Report of Heart and Lung Transplantation. [17] However, these studies largely assessed this population without distinction between mechanism of donation, therefore the isolated impact of conventional cardiac surgery on DCD transplantation has not been thoroughly investigated.

To the best of our knowledge, this is the first national registry study to assess posttransplant outcomes among adult DCD recipients with prior conventional cardiac surgery. In our analysis, conventional cardiac surgery was associated with an over threefold increased risk of 1‐year posttransplant mortality following DCD transplant, paralleling recent clinical trends observing compromised survival in this population more broadly. [13, 17] This emerging pattern reveals the need for reformed approaches to perioperative risk stratification and outcome prediction for recipients with conventional cardiac surgery.

Redo median sternotomy is a well‐established risk factor for postoperative mortality following cardiac surgery, likely driven by altered mediastinal anatomy and dense adhesions that increase operative field complexity and prolong ischemic times. [22, 23] Consistent with this, DCD recipients with prior cardiac surgery in our analysis endured significantly longer graft ischemic times, supporting a mechanistic link between technical complexity and early perioperative vulnerability. Previous studies investigating early outcomes after cardiac transplant have also demonstrated extended duration of cardiopulmonary bypass, longer hospital stays, and higher rates of stroke and renal dysfunction in redo sternotomy patients. [22, 24, 25] Additionally, previous blood transfusions have been hypothesized to stimulate antibody formation and potentiate allograft rejection risk. [22, 26] In our cohort, higher rates of pretransplant blood transfusion among DCD recipients with prior cardiac surgery may reflect greater clinical acuity and increase the risk of early allograft dysfunction or rejection. Collectively, these findings underscore that both technical and patient‐level factors likely contribute to heightened perioperative vulnerability in DCD recipients with prior cardiac surgery. Particularly, longer ischemic times observed among this population emphasize the importance of strategy and communication between the transplanting and procuring surgeon. Future directions aimed at optimizing outcomes for these patients may target refinement in the process of normothermic regional perfusion (NRP) and Organ Care System (OCS) alongside use of advanced preservation practices to mitigate ischemic injury in this high‐risk population.

Patients with valvular pathology constitute a particularly vulnerable patient population, largely due to the substantial burden of progressive pulmonary vascular remodeling. In our cohort, valvular heart failure etiology was more prevalent among DCD recipients with prior cardiac surgery compared to those without, suggesting a higher burden of underlying valvular disease in this population. Pulmonary hypertension (PH) is highly a prevalent complication among patients with advanced mitral and aortic valvular disease and has been previously established as a relative contraindication to heart transplantation given its association with worse postoperative outcomes. [27, 28, 29] Early mortality in recipients with pretransplant PH likely reflect the limited ability of the donor allograft to accommodate elevated pulmonary vascular resistance in the immediate postoperative period. In this context, recipients with prior valvular intervention may be particularly susceptible to adverse outcomes due to underlying, and potentially irreversible, pulmonary vascular injury. Consistent with this hypothesis, DCD recipients with prior valvular repair or replacement in our cohort demonstrated the highest 1‐year mortality among cardiac surgical subtypes. Notably, those undergoing combined CABG and valvular intervention conferred the poorest outcomes, suggesting that cumulative surgical burden may further compound this physiologic risk and be more predicative of adverse posttransplant outcomes than isolated valvular or coronary disease. Further investigation characterizing the presence and/or degree of pulmonary vascular remodeling in this subgroup is warranted to better define the role of valvular disease and prior valvular operative intervention in shaping posttransplant outcomes among DCD recipients.

Rising transplant volumes alongside a growing waitlist underscore the importance of efficient and safe allograft allocation. [3] The adoption of recovering hearts procured after circulatory death has yielded consistently comparable survival compared to DBD, offering a promising approach to safely expand the donor pool. [4, 8, 9] Continued demonstration of similar survival outcomes in DCD and DBD recipients is crucial to validate DCD as a safe, non‐inferior mechanism of donation. Risk‐adjusted analysis of recipients with conventional cardiac surgery demonstrated comparable survival between DCD and DBD transplant. These findings support DCD as a safe and viable alternative to expand the donor pool in this high‐risk population. Moreover, this study solidifies the increased risk of early mortality among recipients with conventional cardiac surgery independent of donor type. This finding should be at the forefront of preoperative physician‐patient counseling, informing postoperative care and rejection management. Linkage of national transplant registries with operative data reports or querying EHRs of multi‐hospital, single healthcare systems may offer a more granular characterization of cardiac surgical history in future investigations, including intervention type, number of prior sternotomies, and intraoperative complications.

### 4.1 Limitations

The retrospective, non‐randomized nature of this study constrains our ability to declare causal conclusions. Our analysis included only adults who underwent isolated heart transplantation, limiting generalizability to pediatric populations and multi‐organ transplantation. In multivariable analysis, the limited number of observed events relative to total covariates may introduce the potential for possible overfitting. Additionally, multicenter databases like UNOS are susceptible to data entry inaccuracies and missing data (i.e. preoperative panel reactive antibody), therefore unmeasured confounding cannot be excluded. Notably, UNOS lacks metrics detailing specific timing, subtype, and number of cardiac operations performed prior to transplantation. Granular procedural detail beyond the predefined surgical subtype categories presented in our analysis is not available in the UNOS database. Poor characterization of cardiac procedures in the “other” subgroup limits accurate interpretation of outcomes and may confound comparisons across surgical subtypes. Findings with respect to the “Other” category as defined by UNOS should therefore be interpreted with caution. Relevant details regarding donor management and advanced preservation techniques (NRP, OCS) are also missing from UNOS, including withdrawal and agonal phases, time to NRP initiation, and trends of hemodynamic parameters including LVEF, cardiac index, and perfusion markers during NRP or OCS. This restricts evaluation of the physiologic quality of DCD allografts and the potential impact of preservation strategies on outcomes.

## 5 Conclusion

Conventional cardiac surgical history, present in nearly one‐third of DCD recipients, is associated with reduced 1‐year survival, notably following valvular interventions. Furthermore, no observed survival difference after multivariable adjustment between DCD and DBD recipients with prior conventional cardiac surgery suggests that DCD donor use does not confer additional mortality risk, supporting the safe and continued expansion of DCD transplant in this high‐risk population. Future studies are necessary to characterize the long‐term impact of conventional cardiac surgery on DCD transplant outcomes and delineate survival risk profiles across surgical subtypes.

## Author Contributions

**Ander Dorken‐Gallastegi**: Critical revision of article, approval of article. **Gavin W. Hickey**: Critical revision of article, approval of article. **Yeahwa Hong**: Concept/design, data analysis/interpretation, drafting article, critical revision of article, approval of article. **Edward T. Horn**: Critical revision of article, approval of article. **Nidhi Iyanna**: Critical revision of article, approval of article. **David J. Kaczorowski**: Concept/design, data analysis/interpretation, drafting article, critical revision of article, approval of article. **Mary E. Keebler**: Critical revision of article, approval of article. **Samantha N. Machinski**: Concept/design, data analysis/interpretation, drafting article, critical revision of article, approval of article. **Umar Nasim**: Critical revision of article, approval of article. **Laura Seese**: Critical revision of article, approval of article. **Brian E. Woolley**: Critical revision of article, approval of article.

## Funding

The authors have nothing to report.

## Disclosure

David Kaczorowski received consultant and speaking fees from Medtronic and Abiomed and research support from Abbott, TransMedics, and XVIVO.

## Conflicts of Interest

The authors declare no conflicts of interest.

## Supporting information




**Supporting Information**: ctr70569‐sup‐0001‐tablesS1‐S4.docx

## Data Availability

Data reported here were supplied by UNOS as the contractor for the Organ Procurement and Transplantation Network. Interpretation and reporting of these data are the responsibility of the author(s) and in no way should be seen as an official policy of or interpretation by the OPTN or U.S. Government.
