# Supplementary material for: Conventional Cardiac Surgery in Donation After Circulatory Death Heart Transplantation: A United Network for Organ Sharing Registry Analysis
Source: Clin Transplant. 2026 May 18;40:e70569. doi: 10.1111/ctr.70569 (PMC13182683; doi:10.1111/ctr.70569)
Supplement: Supplementary file 1 — Supporting Information: ctr70569‐sup‐0001‐tablesS1‐S4.docx [file CTR-40-e70569-s001.docx]

**Table S1.** Baseline recipient, donor, and transplant characteristic missingness.

| **Parameter** | **Missing Data** |
| --- | --- |
| *Recipient Characteristics* | |
| Recipient age (years) | 0 (0.0%) |
| Female gender | 0 (0.0%) |
| Body mass index (kg/m^2^) | 1 (0.1%) |
| Race | 1 (0.1%) |
| Blood Type | 0 (0.0%) |
| Education Level | 18 (2.3%) |
| Functional status | 40 (5.1%) |
| Heart failure etiology | 49 (6.2%) |
| Intensive care unit state at time of transplantation | 0 (0.0%) |
| Pretransplant dialysis | 0 (0.0%) |
| Pretransplant mechanical ventilation | 0 (0.0%) |
| Pretransplant infection | 2 (0.3%) |
| Intravenous inotropes | 0 (0.0%) |
| Waitlist blood transfusion | 0 (0.0%) |
| Cerebrovascular accident | 0 (0.0%) |
| Diabetes mellitus | 0 (0.0%) |
| Hypertension | 84 (10.7%) |
| Positive CMV serology | 0 (0.0%) |
| Total bilirubin (mg/dL) | 2 (0.3%) |
| Serum creatinine (mg/dL) | 0 (0.0%) |
| IABP | 0 (0.0%) |
| ECMO | 0 (0.0%) |
| Impella | 0 (0.0%) |
| Cardiac output (L/min) | 29 (3.7%) |
| Cardiac index (L/min/m2) | 23 (2.9%) |
| Mean pulmonary artery pressure (mmHg) | 22 (2.8%) |
| Mean pulmonary capillary wedge pressure (mmHg) | 46 (5.9%) |
| Mean pulmonary vascular resistance (Wood units) | 75 (9.6%) |
| Transpulmonary Gradient (mmHg) | 68 (8.7%) |
| *Donor Characteristics* | |
| Donor Age | 0 (0.0%) |
| Female gender | 0 (0.0%) |
| Body mass index | 0 (0.0%) |
| Donor race | 0 (0.0%) |
| Donor blood type | 0 (0.0%) |
| Donor mechanism of death | 0 (0.0%) |
| Donor diabetes mellitus | 3 (0.4%) |
| Donor Hepatitis C | 0 (0.0%) |
| Positive CMV serology | 8 (1.0%) |
| Hypertension | 5 (0.6%) |
| Graft LVEF <50% | 2 (0.3%) |
| Total bilirubin (mg/dL) | 0 (0.0%) |
| Serum creatinine (md/dL) | 0 (0.0%) |
| *Transplant Characteristics* | |
| Sex matched | 0 (0.0%) |
| Race matched | 0 (0.0%) |
| HLA matched | 97 (12.4%) |
| ABO matched | 0 (0.0%) |
| CMV status matched | 8 (1.0%) |
| Waitlist time (days) | 0 (0.0%) |
| Distance-recipient distance (Nautical Miles) | 0 (0.0%) |
| Total graft ischemic time (hours) | 1 (0.1%) |
| Waitlist status at listing | 34 (4.3%) |
| Waitlist status at transplantation | 0 (0.0%) |
| *Post-Transplant Complications* | |
| Dialysis | 1 (0.1%) |
| Stroke | 0 (0.0%) |
| Permanent pacemaker implantation | 0 (0.0%) |
| Length of stay (days) | 4 (0.5%) |
| Treated acute rejection | 0 (0.0%) |

**Table S2.** Multivariable Cox Regression Model for 1-year Post-transplant Mortality among Donation After Circulatory Death Heart Transplant Recipients

| Covariate | **Hazard Ratio** | **95% Confidence Interval** | **P-value** |
| --- | --- | --- | --- |
| Prior Cardiac Surgery | 3.26 | 1.71-6.22 | <0.001 |
| Recipient age (years) | 1.02 | 1.00-1.06 | 0.071 |
| Female Gender | 2.21 | 1.06-4.61 | 0.034 |
| Recipient Body Mass Index (kg/m^2^) | 1.02 | 0.95-1.09 | 0.607 |
| Diabetes mellitus | 1.32 | 0.68-2.54 | 0.152 |
| Serum creatinine (mg/dL) | 2.56 | 1.73-3.79 | <0.001 |
| Total bilirubin (mg/dL) | 1.55 | 1.17-2.06 | 0.002 |
| Donor age (years) | 1.00 | 0.96-1.04 | 0.968 |
| Total graft ischemic time (hours) | 1.22 | 1.06-1.41 | 0.005 |
| Total waitlist time (days) | 1.00 | 0.999-1.00 | 0.587 |
| Waitlist Transfusion | 0.84 | 0.34-2.10 | 0.714 |

**Table S3. A)** Baseline recipient, donor, and transplant characteristics stratified by history of prior cardiac surgical in propensity-matched cohort. **B)** Posttransplant outcomes stratified by history of prior cardiac surgery in the propensity-matched cohort.

**A)**

| Parameter | **No Prior Cardiac Surgery**  **(n = 191)** | **Prior Cardiac Surgery**  **(n = 191)** | **P-value** |
| --- | --- | --- | --- |
| *Recipient Characteristics* | | | |
| Recipient age (years) | 60 (52-66) | 60 (50-66) | 0.49 |
| Female gender | 24 (12.6%) | 37 (19.4%) | 0.07 |
| Body mass index (kg/m^2^) | 27.85 (4.53) | 28.24 (4.81) | 0.41 |
| Race |  |  | 0.85 |
| White | 135 (70.7%) | 141 (73.8%) |  |
| Black | 28 (14.7%) | 26 (13.6%) |  |
| Hispanic | 19 (9.9%) | 17 (8.9%) |  |
| Asian | 8 (4.2%) | 5 (2.6%) |  |
| Other | 1 (0.5%) | 2 (1.0%) |  |
| Blood Type |  |  | 0.84 |
| A | 76 (39.8%) | 72 (37.7%) |  |
| AB | 5 (2.6%) | 5 (2.6%) |  |
| B | 23 (12.0%) | 29 (15.2%) |  |
| O | 87 (45.5%) | 85 (44.5%) |  |
| Education Level |  |  | 0.42 |
| High school | 69 (36.1%) | 67 (35.1%) |  |
| College | 105 (55.0%) | 99 (51.8%) |  |
| Graduate degree | 17 (8.9%) | 25 (13.1%) |  |
| Functional status |  |  | 0.30 |
| Independent | 8 (4.2%) | 15 (7.9%) |  |
| Requires Assistance | 95 (49.7%) | 88 (46.1%) |  |
| Hospitalized | 88 (46.1%) | 88 (46.1%) |  |
| Heart failure etiology |  |  | <0.001 |
| Non-Ischemic | 94 (49.2%) | 63 (33.0%) |  |
| Ischemic | 51 (26.7%) | 81 (42.4%) |  |
| Congenital | 0 (0.0%) | 5 (2.6%) |  |
| Restrictive | 15 (7.9%) | 5 (2.6%) |  |
| Valvular | 2 (1.0%) | 17 (8.9%) |  |
| Hypertrophic | 12 (6.3%) | 13 (6.8%) |  |
| Other | 0 (0.0%) | 0 (0.0%) |  |
| Missing | 17 (8.9%) | 7 (3.7%) |  |
| Intensive care unit state at time of transplantation | 66 (34.6%) | 68 (35.6%) | 0.83 |
| Pretransplant dialysis | 1 (0.5%) | 2 (1.0%) | 0.56 |
| Pretransplant mechanical ventilation | 1 (0.5%) | 2 (1.0%) | 0.56 |
| Pretransplant infection | 5 (2.6%) | 8 (4.2%) | 0.41 |
| Intravenous inotropes | 62 (32.5%) | 67 (35.1%) | 0.59 |
| Waitlist blood transfusion | 33 (17.3%) | 37 (18.8%) | 0.78 |
| Cerebrovascular accident | 16 (8.4%) | 18 (9.4%) | 0.72 |
| Diabetes mellitus | 70 (36.6%) | 67 (35.1%) | 0.75 |
| Hypertension | 52 (29.9%) | 47 (27.8%) | 0.67 |
| Positive CMV serology | 102 (53.4%) | 96 (50.3%) | 0.54 |
| Total bilirubin (mg/dL) | 0.7 (0.5-1.1) | 0.6 (0.4-1.0) | 0.09 |
| Serum creatinine (mg/dL) | 1.2 (1.0-1.5) | 1.16 (0.94-1.4) | 0.46 |
| IABP | 40 (20.9%) | 34 (17.8%) | 0.44 |
| ECMO | 4 (2.1%) | 3 (1.6%) | 0.65 |
| Cardiac output (L/min) | 4.41 (1.36) | 4.29 (1.219) | 0.35 |
| Cardiac index (L/min/m2) | 2.10 (0.65) | 2.02 (0.48) | 0.15 |
| Mean pulmonary artery pressure (mmHg) | 27.95 (10.34) | 26.82 (10.64) | 0.30 |
| Mean pulmonary capillary wedge pressure (mmHg) | 19.11 (8.80) | 17.49 (7.88) | 0.07 |
| Mean pulmonary vascular resistance (Wood units) | 2.36 (1.53) | 2.32 (1.45) | 0.80 |
| Transpulmonary Gradient (mmHg) | 9.46 (5.14) | 9.29 (5.02) | 0.75 |
| *Donor Characteristics* | | | |
| Donor Age | 33 (26-38) | 31 (24-38) | 0.42 |
| Female gender | 23 (12.0%) | 30 (15.7%) | 0.30 |
| Body mass index | 27.31 (5.95) | 27.55 (5.68) | 0.69 |
| Donor race |  |  | 0.88 |
| White | 143 (74.9%) | 145 (75.9%) |  |
| Black | 15 (7.9%) | 17 (8.9%) |  |
| Hispanic | 23 (12.0%) | 21 (11.0%) |  |
| Asian | 3 (1.6%) | 4 (2.1%) |  |
| Other | 7 (3.7%) | 4 (2.1%) |  |
| Donor blood type |  |  | 0.76 |
| A | 68 (35.6%) | 66 (34.6%) |  |
| AB | 1 (0.5%) | 0 (0.0%) |  |
| B | 16 (8.4%) | 18 (9.4%) |  |
| O | 106 (55.5%) | 107 (56.0%) |  |
| Donor mechanism of death |  |  | 0.78 |
| Trauma | 90 (47.1%) | 81 (42.4%) |  |
| Cardiovascular | 18 (9.4%) | 19 (9.9%) |  |
| Drug overdose | 39 (20.4%) | 46 (24.1%) |  |
| Other | 44 (23.0%) | 45 (23.6%) |  |
| Donor diabetes mellitus | 7 (3.7%) | 7 (3.7%) | 1.00 |
| Donor Hepatitis C | 17 (8.9%) | 18 (9.4%) | 0.86 |
| Positive CMV serology | 117 (61.3%) | 108 (56.5%) | 0.35 |
| Hypertension | 31 (16.2%) | 27 (14.1%) | 0.47 |
| Graft LVEF | 61 (59-68) | 64 (59-67) | 0.08 |
| Total bilirubin (mg/dL) | 0.6 (0.4-1.0) | 0.6 (0.4-0.9) | 0.22 |
| Serum creatinine (md/dL) | 0.78 (0.6-1.03) | 0.77 (0.6-1.01) | 0.81 |
| *Transplant Characteristics* | | | |
| Sex matched | 160 (83.8%) | 171 (86.8%) | 0.39 |
| Race matched | 110 (57.6%) | 120 (60.9%) | 0.40 |
| HLA matched | 17 (8.9%) | 20 (10.2%) | 0.73 |
| ABO matched | 168 (88.0%) | 170 (86.3%) | 0.54 |
| CMV status matched | 108 (56.5%) | 97 (49.2%) | 0.18 |
| Waitlist time (days) | 21 (6-111) | 34 (13-163) | 0.02 |
| Distance-recipient distance (Nautical Miles) | 305 (108-536) | 317 (135-569) | 0.44 |
| Total graft ischemic time (hours) | 4.7 (3.4-6.2) | 5.3 (3.7-6.5) | 0.26 |
| Waitlist status at listing |  |  | 0.76 |
| 1 | 3 (1.7%) | 3 (1.6%) |  |
| 2 | 40 (22.1%) | 34 (18.6%) |  |
| 3 | 13 (7.2%) | 16 (8.7%) |  |
| 4 | 47 (26.0%) | 56 (30.6%) |  |
| 5 | 1 (0.6%) | 0 (0.0%) |  |
| 6 | 77 (42.5%) | 74 (40.4%) |  |
| Waitlist status at transplantation |  |  | 0.46 |
| 1 | 5 (2.6%) | 5 (2.6%) |  |
| 2 | 76 (39.8%) | 70 (36.6%) |  |
| 3 | 15 (7.9%) | 17 (8.9%) |  |
| 4 | 43 (22.5%) | 58 (30.4%) |  |
| 5 | 1 (0.5%) | 0 (0.0%) |  |
| 6 | 51 (26.7%) | 41 (21.5%) |  |

Abbreviations: CMV, cytomegalovirus; *ECMO*, extracorporeal membrane oxygenation; HLA, human leukocyte antigen; IABP, intra-aortic balloon pump; ICU, intensive care unit; LVEF, left ventricular ejection fraction.

**B)**

| **Complication** | **No Prior Cardiac Surgery**  **(n = 191)** | **Prior Cardiac Surgery**  **(n = 191)** | **P-value** |
| --- | --- | --- | --- |
| Dialysis | 39 (20.4%) | 44 (23.2%) | 0.52 |
| Stroke | 6 (3.1%) | 11 (5.8%) | 0.21 |
| Permanent pacemaker implantation | 4 (2.1%) | 2 (1.0%) | 0.41 |
| Length of stay (days) | 16 (12-25) | 16 (12-27) | 0.71 |
| Treated acute rejection | 13 (6.8%) | 12 (6.3%) | 0.84 |
| **Cause of Death** | **No Prior Cardiac Surgery**  **(n = 14)** | **Prior Cardiac Surgery**  **(n = 33)** | **P-value** |
| Graft Failure | 2 (14%) | 4 (12%) | 0.76 |
| Infection | 4 (29%) | 8 (24%) |  |
| Cardiovascular | 1 (7%) | 3 (9%) |  |
| Pulmonary | 1 (7%) | 1 (3%) |  |
| Cerebrovascular | 0 (0%) | 6 (18%) |  |
| Hemorrhage | 0 (0%) | 0 (0%) |  |
| Malignancy | 1 (7%) | 4 (12%) |  |
| Other, misc. | 4 (29%) | 6 (18%) |  |
| Unknown/not reported | 1 (7%) | 1 (3%) |  |

**Table S4.** Multivariable Cox Regression Model for 1-year Post-transplant Mortality in Heart Transplant Recipients with Prior Cardiac Surgical History

| Covariate | **Hazard Ratio** | **95% Confidence Interval** | **P-value** |
| --- | --- | --- | --- |
| Donation After Circulatory Death Transplant | 0.897 | 0.57-1.40 | 0.635 |
| Recipient age (years) | 1.01 | 1.00-1.03 | 0.017 |
| Female gender | 1.18 | 0.91-1.55 | 0.216 |
| Diabetes mellitus | 1.01 | 0.79-1.28 | 0.934 |
| Serum creatinine (mg/dL) | 1.11 | 1.03-1.19 | 0.004 |
| Total bilirubin (mg/dL) | 1.07 | 1.04-1.11 | <0.001 |
| Heart failure etiology |  |  |  |
| Non-Ischemic | Ref | Ref | Ref |
| Ischemic | 1.24 | 0.95-1.61 | 0.114 |
| Congenital | 0.66 | 0.21-2.11 | 0.481 |
| Restrictive | 1.68 | 1.00-2.82 | 0.049 |
| Valvular | 1.21 | 0.66-2.21 | 0.540 |
| Hypertrophic | 0.60 | 0.29-1.23 | 0.161 |
| Donor age (years) | 1.00 | 1.00-1.01 | 0.820 |
| Total graft ischemic time (hours) | 1.08 | 1.00-01.18 | 0.058 |
| Total waitlist time (days) | 1.00 | 1.00-1.0003 | 0.371 |
| Waitlist Transfusion | 1.07 | 0.82-1.40 | 0.632 |
